# Supplementary material for: Genome-wide analysis of long noncoding RNAs, 24-nt siRNAs, DNA methylation and H3K27me3 marks in Brassica rapa
Source: PLoS One. 2021 Mar 31;16(3):e0242530. doi: 10.1371/journal.pone.0242530 (PMC8011741; doi:10.1371/journal.pone.0242530)
Supplement: S1 Fig — Log2 FPKM of mRNAs represented in blue (positive values) and orange (negative values), and lncRNAs in green (positive values) and pink (negative values). (PPTX) [file pone.0242530.s001.pptx]

## Slide 1
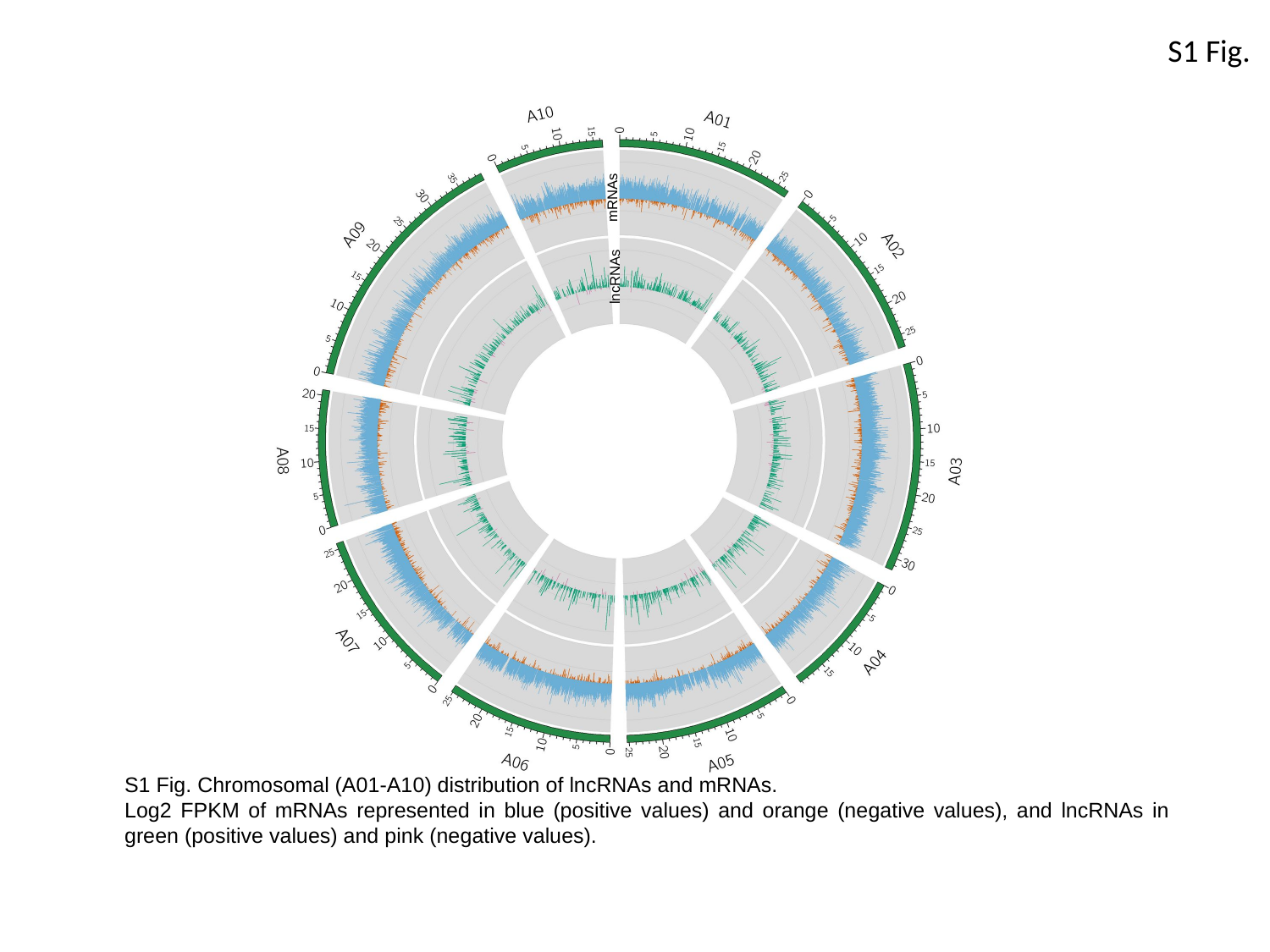

S1 Fig.
mRNAs
lncRNAs
S1 Fig. Chromosomal (A01-A10) distribution of lncRNAs and mRNAs.
Log2 FPKM of mRNAs represented in blue (positive values) and orange (negative values), and lncRNAs in green (positive values) and pink (negative values).
