# Supplementary material for: Genome-wide analysis of long noncoding RNAs, 24-nt siRNAs, DNA methylation and H3K27me3 marks in Brassica rapa
Source: PLoS One. 2021 Mar 31;16(3):e0242530. doi: 10.1371/journal.pone.0242530 (PMC8011741; doi:10.1371/journal.pone.0242530)
Supplement: S2 Fig — “Total” represents the DNA methylation level of all regions. (PPTX) [file pone.0242530.s002.pptx]

## Slide 1
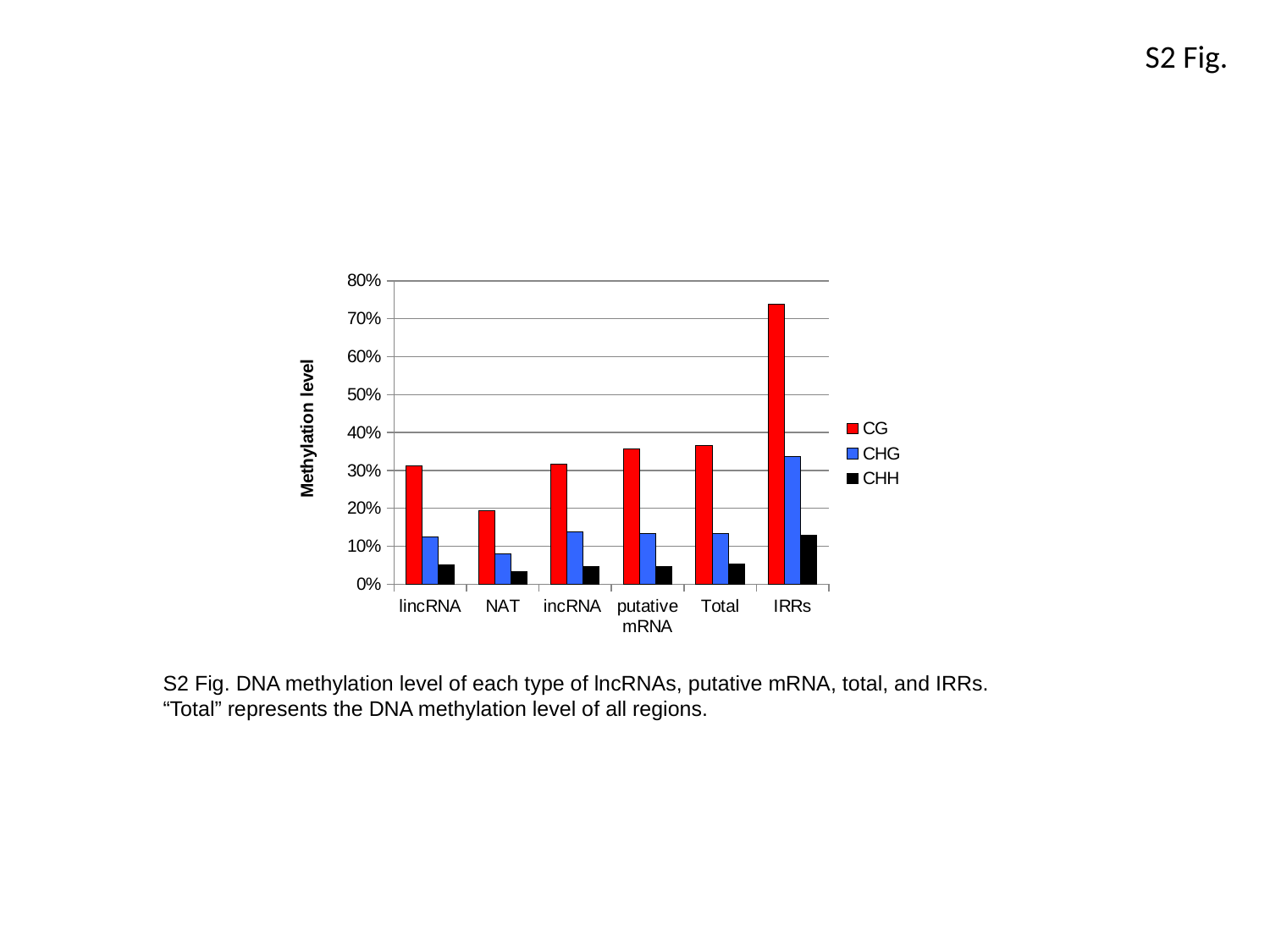

S2 Fig.
### Chart
| Category | CG | CHG | CHH |
|---|---|---|---|
| lincRNA | 0.311433 | 0.124301 | 0.0500086 |
| NAT | 0.193772 | 0.0804019 | 0.033373 |
| incRNA | 0.316215 | 0.137579 | 0.0473071 |
| putative mRNA | 0.356343 | 0.132879 | 0.0475826 |
| Total | 0.365392386244115 | 0.134425472817307 | 0.0534067285186912 |
| IRRs | 0.737345552168316 | 0.337588012135657 | 0.129893344852088 |S2 Fig. DNA methylation level of each type of lncRNAs, putative mRNA, total, and IRRs. “Total” represents the DNA methylation level of all regions.
