# Supplementary material for: Genome-wide analysis of long noncoding RNAs, 24-nt siRNAs, DNA methylation and H3K27me3 marks in Brassica rapa
Source: PLoS One. 2021 Mar 31;16(3):e0242530. doi: 10.1371/journal.pone.0242530 (PMC8011741; doi:10.1371/journal.pone.0242530)
Supplement: S3 Fig — (PPTX) [file pone.0242530.s003.pptx]

## Slide 1
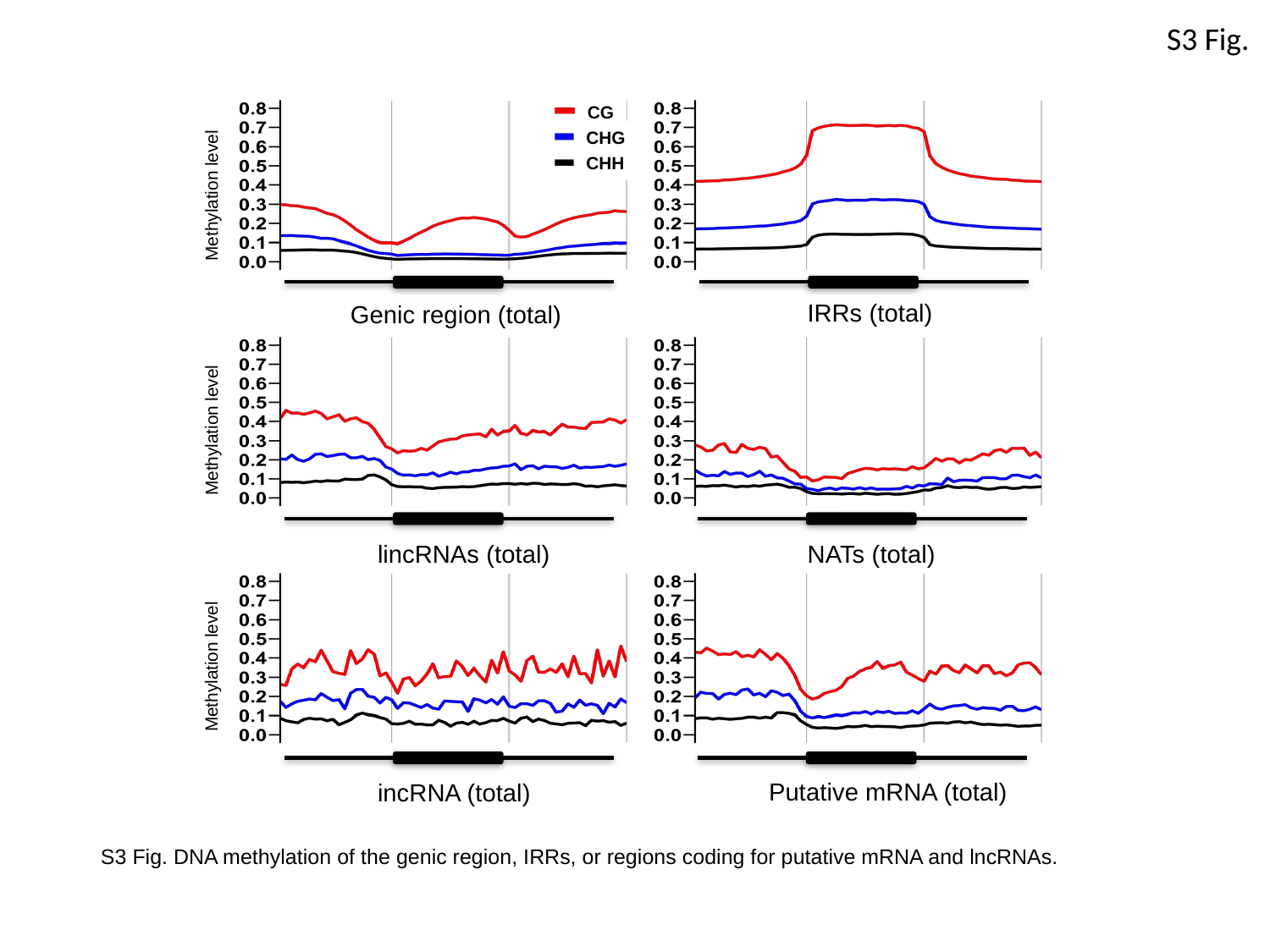

S3 Fig.
CG
CHG
CHH
Methylation level
IRRs (total)
Genic region (total)
Methylation level
lincRNAs (total)
NATs (total)
Methylation level
Putative mRNA (total)
incRNA (total)
S3 Fig. DNA methylation of the genic region, IRRs, or regions coding for putative mRNA and lncRNAs.
