# Supplementary material for: Genome-wide analysis of long noncoding RNAs, 24-nt siRNAs, DNA methylation and H3K27me3 marks in Brassica rapa
Source: PLoS One. 2021 Mar 31;16(3):e0242530. doi: 10.1371/journal.pone.0242530 (PMC8011741; doi:10.1371/journal.pone.0242530)
Supplement: S4 Fig — (PPTX) [file pone.0242530.s004.pptx]

## Slide 1
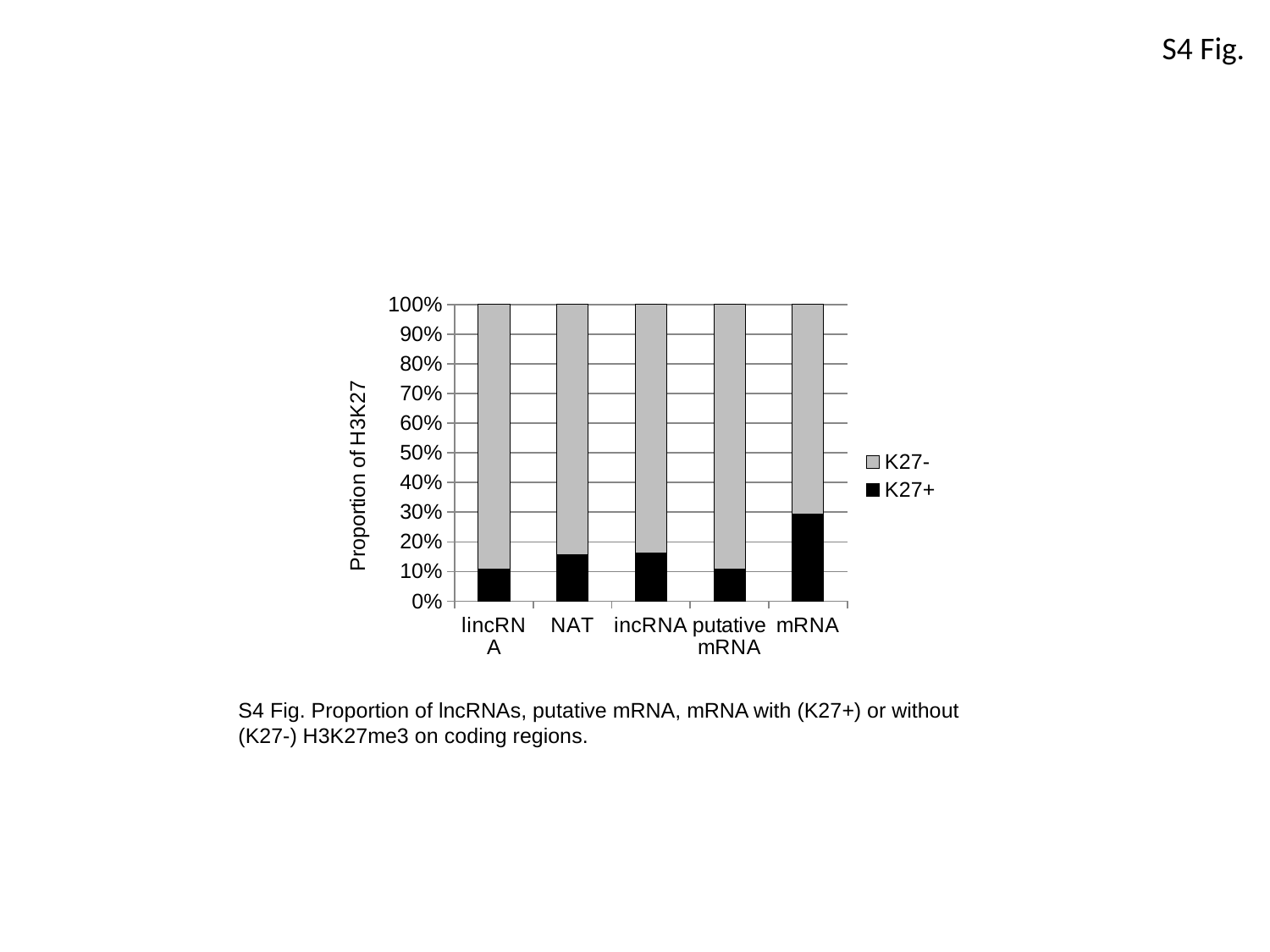

S4 Fig.
### Chart
| Category | K27+ | K27- |
|---|---|---|
| lincRNA | 127.0 | 1046.0 |
| NAT | 83.0 | 446.0 |
| incRNA | 15.0 | 77.0 |
| putative mRNA | 53.0 | 437.0 |
| mRNA | 11655.0 | 27954.0 |S4 Fig. Proportion of lncRNAs, putative mRNA, mRNA with (K27+) or without (K27-) H3K27me3 on coding regions.
