# Supplementary material for: Genome-wide analysis of long noncoding RNAs, 24-nt siRNAs, DNA methylation and H3K27me3 marks in Brassica rapa
Source: PLoS One. 2021 Mar 31;16(3):e0242530. doi: 10.1371/journal.pone.0242530 (PMC8011741; doi:10.1371/journal.pone.0242530)
Supplement: S5 Fig — (PPTX) [file pone.0242530.s005.pptx]

## Slide 1
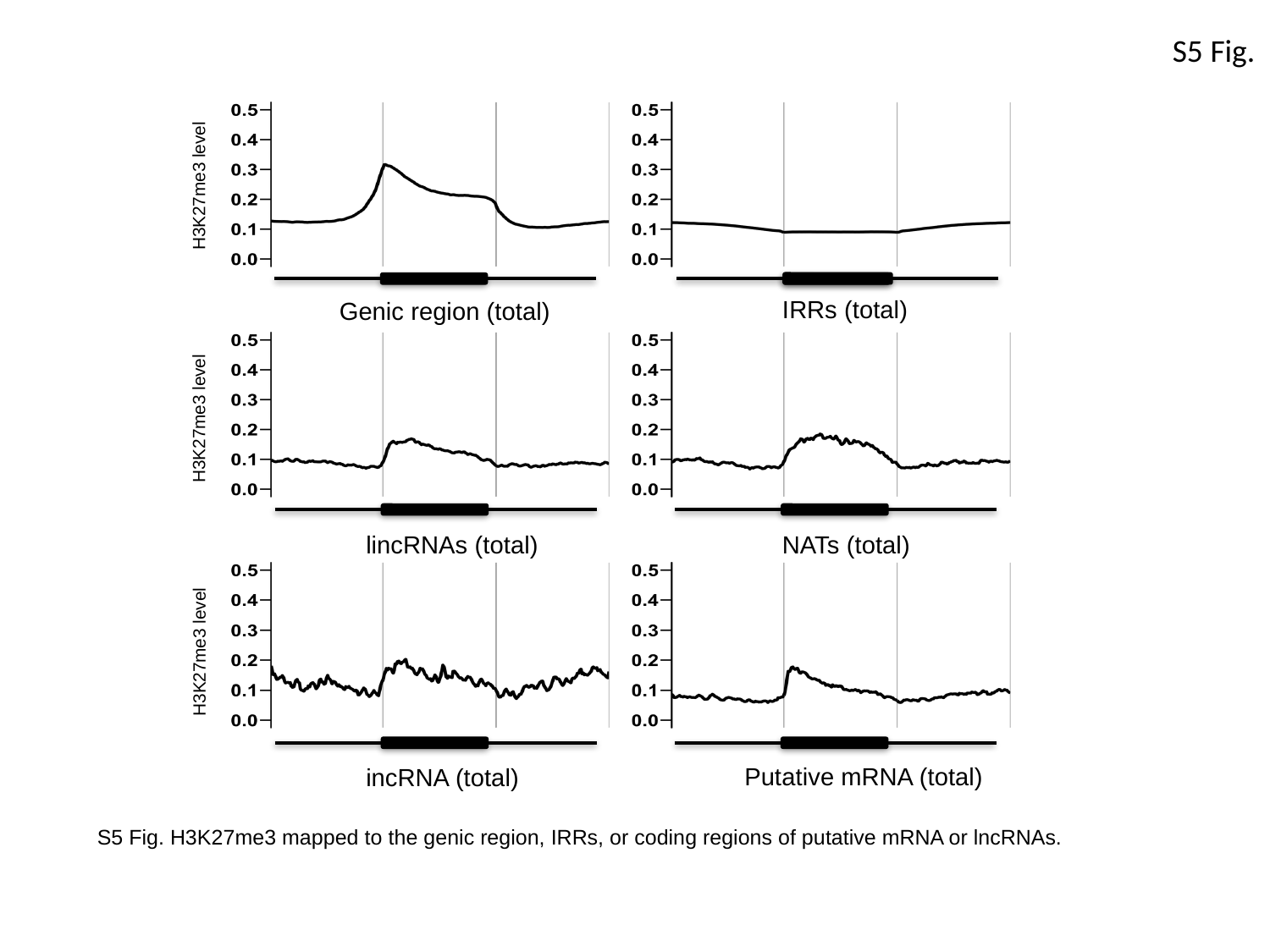

S5 Fig.
H3K27me3 level
IRRs (total)
Genic region (total)
H3K27me3 level
lincRNAs (total)
NATs (total)
H3K27me3 level
Putative mRNA (total)
incRNA (total)
S5 Fig. H3K27me3 mapped to the genic region, IRRs, or coding regions of putative mRNA or lncRNAs.
