# Supplementary material for: Genome-wide analysis of long noncoding RNAs, 24-nt siRNAs, DNA methylation and H3K27me3 marks in Brassica rapa
Source: PLoS One. 2021 Mar 31;16(3):e0242530. doi: 10.1371/journal.pone.0242530 (PMC8011741; doi:10.1371/journal.pone.0242530)
Supplement: S6 Fig — The number represents each line as follows: For B. rapa lines, 1 = RJKB-T24, 2 = Homei, 3 = Harunosaiten, 4 = BRA2209, 5 = Osome, 6 = Yellow Sarson. For B. oleracea lines, 7 = Reiho, 8 = Matsunami, 9 = Kinkei 201. (PPTX) [file pone.0242530.s006.pptx]

## Slide 1
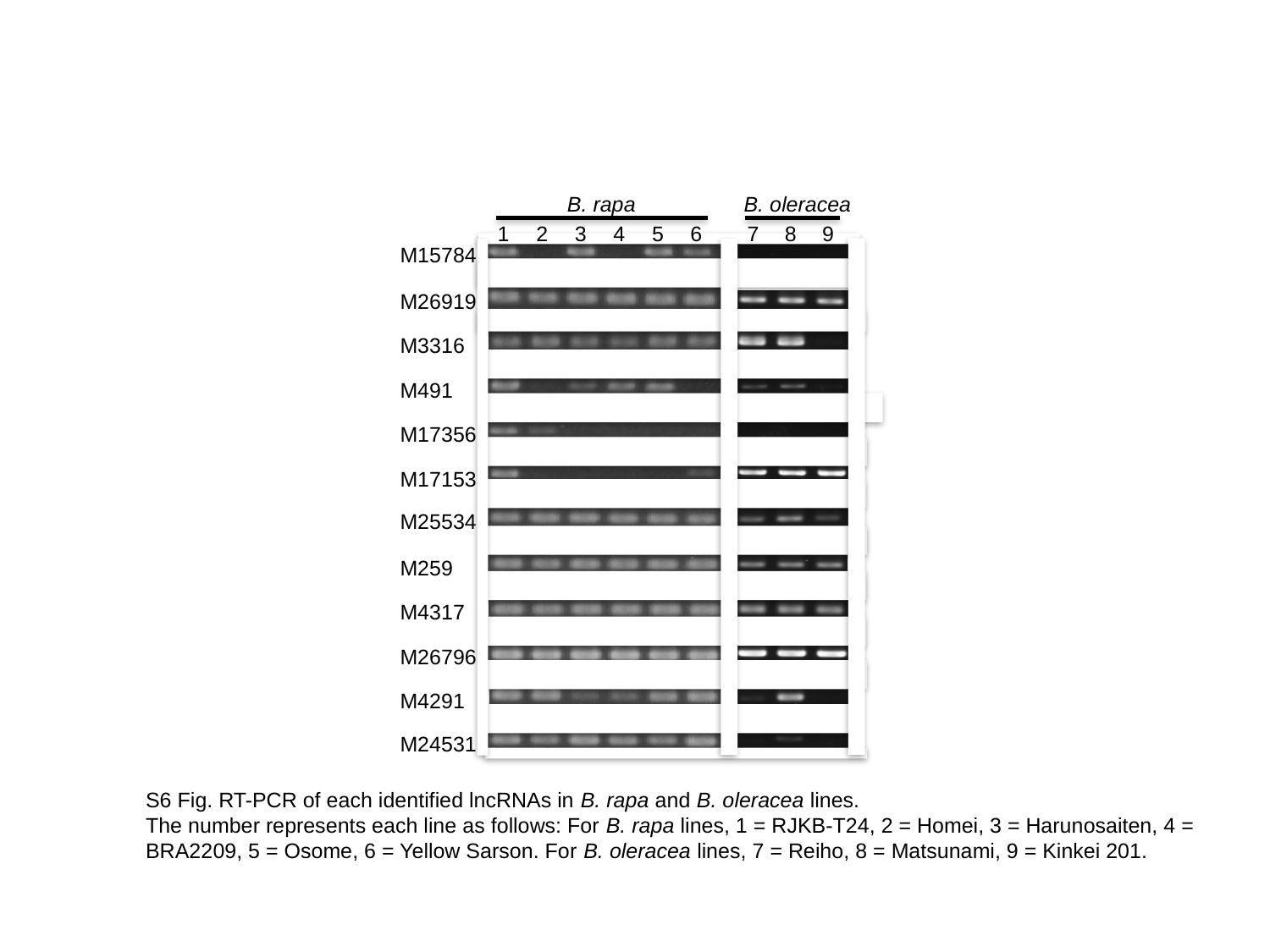

B. rapa
B. oleracea
1
2
3
4
5
6
7
8
9
M15784
M26919
M3316
M491
M17356
M17153
M25534
M259
M4317
M26796
M4291
M24531
S6 Fig. RT-PCR of each identified lncRNAs in B. rapa and B. oleracea lines.
The number represents each line as follows: For B. rapa lines, 1 = RJKB-T24, 2 = Homei, 3 = Harunosaiten, 4 = BRA2209, 5 = Osome, 6 = Yellow Sarson. For B. oleracea lines, 7 = Reiho, 8 = Matsunami, 9 = Kinkei 201.
